# The role of genetic polymorphisms in *STIM1* and *ORAI1* for erythropoietin resistance in patients with renal failure

**DOI:** 10.1097/MD.0000000000025243

**Published:** 2021-04-30

**Authors:** Chih-Chin Kao, Henry Sung-Ching Wong, Yu-Jia Wang, Wan-Hsuan Chou, Dyah Aryani Perwitasari, Mai-Szu Wu, Wei-Chiao Chang

**Affiliations:** a Graduate Institute of Clinical Medicine, College of Medicine, Taipei Medical University; b Division of Nephrology, Department of Internal Medicine, Taipei Medical University Hospital; c Division of Nephrology, Department of Internal Medicine, School of Medicine, College of Medicine, Taipei Medical University; d TMU Research Center of Urology and Kidney (TMU-RCUK); e Department of Clinical Pharmacy, School of Pharmacy, Taipei Medical University; f Master Program for Clinical Pharmacogenomics and Pharmacoproteomics, School of Pharmacy, Taipei Medical University; g Ph.D. Program for Neural Regenerative Medicine, College of Medical Science and Technology, Taipei Medical University and National Health Research Institutes, Taipei, Taiwan.; h Faculty of Pharmacy, University of Ahmad Dahlan, Yogyakarta, Indonesia; i Division of Nephrology, Department of Internal Medicine, Shuang Ho Hospital, Taipei Medical University, New Taipei City; j Integrative Research Center for Critical Care, Wan Fang Hospital, Taipei Medical University, Taipei; k Department of Medical Research, Shuang Ho Hospital, Taipei Medical University, New Taipei City, Taiwan.

**Keywords:** erythropoietin resistance, genetic polymorphisms, *ORAI1*, renal failure, *STIM1*, store-operated calcium channel

## Abstract

Anemia is a common complication in patients with renal failure. While erythropoietin is commonly used to treat anemia, some patients exhibit a poor response to erythropoietin. Since store-operated calcium channel (SOC) signaling is one of the erythropoietin activated pathways, we aimed to investigate the association between the genetic polymorphisms of SOC signaling pathway and erythropoietin resistance in patients with renal failure.

Four tagging single nucleotide polymorphisms in *STIM1* and five in *ORAI1* were selected in this study. Genotyping was performed with the TaqMan Allelic Discrimination assay and the association of individual tagging single nucleotide polymorphisms with erythropoietin resistance was analyzed by multivariable adjusted random intercepts model.

194 patients were enrolled in this study. The mean age of participants is 68 years, and 56% were men. The mean erythropoietin resistance index was 9.04 ± 4.51 U/Kg/week/g/dL. We found that patients with the AA genotype of rs1561876 in *STIM1*, and the CC or CT genotypes of rs6486795 in *ORAI1*, were associated with increased risk of erythropoietin resistance. Functional annotation of expression quantitative trait loci revealed that the AA genotype of rs1561876 in *STIM1* has a relatively lower expression of ribonucleotide reductase catalytic subunit M1 in skeletal muscle, while the CC genotype of rs6486795 in *ORAI1* has a relatively higher expression of *ORAI1* in the whole blood and thyroid.

Overall, we demonstrate a significant association between erythropoietin resistance and genetic polymorphisms of *STIM1* and *ORAI1*. Annotation prediction revealed the importance of SOC-mediated calcium signaling for erythropoietin resistance.

## 1 Introduction

Anemia is a major complication in dialysis patients which was associated with poor clinical outcomes such as reduced quality of life, risk of cardiovascular disease, cognitive impairment, and mortality.^[1]^ Since the introduction of recombinant human erythropoietin (EPO) in 1990s, the prognosis for anemia in renal failure has improved drastically. While EPO therapy has become a mainstay treatment under these circumstances, a positive response to EPO is not universal. In fact, a wide variation in EPO dosing was found in the United States Renal Data System. Approximately 10% of patients who had poor responses to EPO were associated with increased risk of death.^[2,3]^ In general, EPO resistance is defined as hemoglobin < 11 g/dL despite a weekly dose of EPO more than 500 IU/kg or 30000 IU/wk.^[4]^

The pathophysiological causes of EPO resistance include chronic inflammation, iron deficiency, blood loss, malnutrition, and hyperparathyroidism.^[2]^ The underlying mechanisms include limited iron availability, low EPO receptor expression, and disrupted EPO signal transduction. Moreover, inflammation has been reported to antagonize EPO activity by the inhibition of EPO receptors and subsequent signaling pathways.^[5]^ However, these mechanisms might not fully explain EPO resistance in patients with renal failure. The role of immune regulation has not been understood well in the pathogenesis of EPO resistance, though Wong HS et al showed that the utilization of “V or J” regions may modify EPO responses in patients with renal failure.^[6]^ Genetic predispositions were also responsible for the EPO response. Several lines of evidence suggest an association between genetic polymorphisms and EPO resistance.^[7,8]^ Polymorphisms of angiotensin-converting enzyme (*ACE*) insertion/deletion was associated with EPO responsiveness in peritoneal dialysis patients, and hemodialysis patients with polymorphisms of *IL-6* needed higher doses of EPO.^[8]^ In addition, polymorphisms of -449G/C and -1151A/C in *DDAH2* gene may influence EPO resistance and plasma asymmetric dimethylarginine concentration in hemodialysis patients.^[9]^ In a Korean cohort stuty, polymorphisms in *IL-1B* and *ACE* are significantly associated with the erythropoietin resistance index (ERI).^[10]^ IL-1B encodes a pro-inflammatory cytokine, and ACE protein converts angiotensin I to angiotensin II, which activate the proliferation of erythroid progenitors. The above findings implicated a potential critical role of inflammation in EPO response.

EPO is also known to induce proliferation and differentiation of erythroid cells by increasing of intracellular calcium signaling.^[11,12]^ Among calcium channel types, store-operated calcium channels (SOCs) are prominent in the non-excitable cells. SOC is activated by depletion of Ca^2+^ from the endoplasmic reticulum.^[13,14]^ Since it is constituted of a signaling pathway of EPO activation, whether the polymorphisms of SOC is associated with EPO resistance is not known, therefore, we aimed to investigated the association between genetic polymorphisms of two genes (*STIM1, ORAI1*) in SOC and EPO sensitivity in dialysis patients. We hypothesized that genetic polymorphisms of SOC signaling play a functional role in determining EPO sensitivity.

## 2 Materials and methods

### 2.1 Patient eligibility and enrollment

In this study, 194 patients were enrolled from Taipei Medical University Hospital. All eligible subjects were at or above age 18 at the time of enrollment. All participants had been receiving hemodialysis thrice weekly or peritoneal dialysis for at least 3 months. The blood samples and clinical data were collected from patients at the enrollment. This study was conducted in compliance with the Declaration of Helsinki and the Guidelines for Good Clinical Practice of the International Conference on Harmonization. This study was approved by the Institutional Review Board of Taipei Medical University (no. 201309026). Written informed consents were obtained from all patients.

### 2.2 Study subjects

Patients’ demographics and clinical profiles were collected, including age, gender, dialysis modality, comorbidities, and biochemistry data at enrollment. The response to EPO was evaluated by using the ERI. For ERI profiles, we performed hemogram testing once monthly before dialysis for consecutive 3 months, and collected the EPO dose once monthly for the 3 consecutive months. The equivalent doses of different erythropoietin stimulating agents (ESA) were calculated according to previous report: the conversion ratio was 6000 IU/wk epoietin beta; 30 μg/wk darbepoetin alfa; 100 μg/mo continuous erythropoietin receptor activator.^[15,16]^ The ERI is calculated by dividing the weekly body-weight-adjusted epoetin dose by the hemoglobin concentration (IU/kg/dL/g).^[17,18]^

### 2.3 Selection of candidate tagging SNPs and genotyping

**Figure 1:**
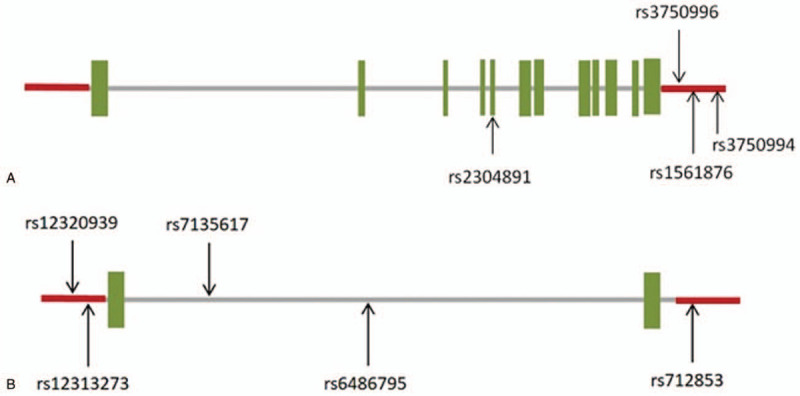
(A) Graphic view of the genotyped human *STIM1* gene. Three polymorphisms were identified in the 3-prime untranslated region ( -UTR), whereas the other one was identified in the exon region. (B) Graphic view of the genotyped human *ORAI1* gene. Two polymorphisms were identified in the 5-prime untranslated region (5’-UTR), two in the intronic region, and one in the 3’-UTR region.

The 4 tagging SNPs (tSNPs) of *STIM1* (rs2304891, rs1561876, rs3750994, and rs3750996; Fig. 1A) and 5 of *ORAI1* (rs712853, rs12313273, rs12320939, rs6486795, and rs7135617; Fig. 1B) were selected, all with a minor allele frequency of > 10% in a Han Chinese in Beijing (CHB) population (http://hapmap.ncbi.nlm.nih.gov/). Genotyping for these tSNPs was performed using the TaqMan Allelic Discrimination Assay (Applied Biosystems, Foster City, CA). Polymerase chain reaction was carried out with an ABI StepOnePlus Thermal Cycler (Applied Biosystems). The fluorescence from different probes was detected and analyzed via the System SDS software version 2.2.2 (Applied Biosystems).

### 2.4 Statistical analysis

Analysis of the association between genetic polymorphisms and ERI level was conducted by mixed-effect regression under a random-intercept model. The beta coefficient of the genotype effect was calculated, and standard error was estimated using a sandwich estimator with bias-reduced linearization adjustment. Meanwhile, covariates adjustment including age, gender, comorbidities (diabetes mellitus and hypertension), iron profiles (ferritin, iron, and total iron-binding capacity), and albumin was performed. Hardy-Weinberg equilibrium of SNPs was evaluated using a chi-squared test. In addition, linkage disequilibrium to identify haplotype blocks of *STIM1* and *ORAI1* were inferred by Haploview 4.2 software (Broad Institute, Cambridge, MA). The haplotypes with a frequency greater than 0.01 were subjected to haplotyping analysis based on a mixed-effect linear model with the same covariates adjustment as above. All analyses used R 3.2.0 (http://www.r-project.org/; http://cran.r-project.org/), and the 2-sided statistical significance level was 0.05.

### 2.5 SNP functional annotation

To investigate the SNP function in the interesting traits and regulatory elements, we queried the candidate genes via the GTEx portal (https://www.gtexportal.org/home/). Meanwhile, we retrieved the significant SNPs tissue expression quantitative trait loci (eQTLs) by genotype discrimination, and these *cis*-eQTLs, defined as 1 Mb in distance between the target SNP position and the probe midpoint for gene expression.^[19]^ In addition, we elucidated the SNP function via SNPNexus (https://www.snp-nexus.org/). It provides the enrichment of polymorphisms in several human genome reference systems with new annotation categories.

## 3 Results

### 3.1 Patient characteristics

A total of 194 patients were enrolled in the study. The mean age of participants was 68 ± 13 years and 55.7% were male (Table 1). The vast majority (90.7%) of patients received regular hemodialysis, while others were peritoneal dialysis patients. The mean ERI was 9.04 ± 4.51 (IU/kg/dL/g). Hardy-Weinberg equilibrium filter were applied for genotyped tSNPs (Supplemental Digital Content, Table S1). We found that the minor allele frequency of these tSNPs in our study were similar to those found in the Asian population from the Genome Aggregation Database (gnomAD) and Taiwan Biobank (TWB) database (Table 2). The results indicated the reliability of our genotyping data.

**Table 1:** Demographics of 194 Taiwanese ESRD patients.

Characteristics	Patients no. (%)
Age (yr)^∗^	68.1 ± 13.0
Gender	
Male	108 (55.67%)
Female	86 (44.33%)
Diabetes Mellitus (DM)	
Yes	95 (48.97%)
No	99 (51.03%)
Hypertension (HTN)	
Yes	163 (84.02%)
No	31 (15.98%)
Dialysis modality	
Hemodialysis (HD)	176 (90.72%)
Peritoneal dialysis (PD)	18 (9.28%)
Erythropoietin resistance index (ERI)^∗^ (U/Kg/wk/g/dL)	9.04 ± 4.51
Albumin^∗^ (g/dL)	4.0 ± 0.4
Ferritin^∗^ (ng/mL)	445.3 ± 540.3
Iron^∗^ (ug/dL)	66.9 ± 26.8
TIBC^∗^ (ug/dL)	238.7 ± 46.4

**Table 2:** Minor allele frequencies of selected tagging single-nucleotide polymorphisms (tSNPs).

Gene	Position (GRCh38.p12)	SNP	Location	Ref	Alt	AFR freq	AMR freq	ASN freq	EUR freq	TWB freq	Our freq
*STIM1*	Chr11:4082294	rs2304891	Syn.variant	A	G	0.16	0.48	0.44	0.53	0.43	0.46
	Chr11:4092165	rs1561876	3’ UTR	A	G	0.62	0.24	0.25	0.12	0.27	0.30
	Chr11:4092240	rs3750994	3’ UTR	T	G	0.01	0.15	0.20	0.03	0.21	0.20
	Chr11:4091970	rs3750996	3’ UTR	A	G	0.00	0.00	0.22	0.00	0.22	0.24
*ORAI1*	Chr12:121641762	rs712853	3’ UTR	A	G	0.59	0.19	0.33	0.28	0.32	0.32
	Chr12:121625105	rs12313273	Intergenic	T	C	0.29	0.33	0.29	0.18	0.30	0.26
	Chr12:121624817	rs12320939	Intergenic	T	G	0.31	0.53	0.52	0.58	0.51	0.48
	Chr12:121638011	rs6486795	Intron	T	C	0.34	0.35	0.37	0.20	0.38	0.38
	Chr12:121631099	rs7135617	Intron	G	T	0.10	0.48	0.41	0.56	0.42	0.41

### 3.2 Associations of *STIM1* and *ORAI1* polymorphisms with ERI

After multivariable adjustments for potential confounding covariates, we found that minor allele of rs1561876 in *STIM1* was associated with a lower risk of EPO resistance in a dominant model (GG/GA vs. AA; beta coefficient: -0.691; *P* = 0.024) (Table 3). In addition, the minor allele of rs6486795 in *ORAI1* was also associated with a higher risk of EPO resistance in a dominant model (CC/CT vs. TT; beta coefficient: 0.676; *P* = 0.029) (Table 4).

**Table 3:** Associations between *STIM1* tSNPs and erythropoietin resistant index (ERI).

SNP	Major	Minor	MAF^†^	Model	Beta Coeff.^‡^	Std. Err.^§^	*P*-value^d^
rs2304891	A	G	0.460	Additive	0.156	0.184	.397
				Dominant	0.016	0.290	.957
				Recessive	0.446	0.342	.193
rs1561876	A	G	0.299	Additive	−0.344	0.230	.137
				Dominant	−0.691	0.304	**.024**^∗^
				Recessive	0.259	0.487	.595
rs3750994	T	G	0.203	Additive	−0.241	0.266	.365
				Dominant	−0.481	0.306	.117
				Recessive	0.755	0.700	.282
rs3750996	A	G	0.237	Additive	−0.016	0.227	.944
				Dominant	0.099	0.299	.741
				Recessive	−0.420	0.517	.417

**Table 4:** Associations between *ORAI1* tSNPs and erythropoietin resistant index (ERI).

SNP	Major	Minor	MAF^†^	Model	Beta Coeff.^‡^	Std. Err.^§^	*P*-value^d^
rs712853	A	G	0.316	Additive	−0.016	0.184	.931
				Dominant	−0.054	0.292	.855
				Recessive	0.053	0.325	.870
rs12313273	T	C	0.257	Additive	−0.141	0.202	.485
				Dominant	−0.230	0.308	.456
				Recessive	−0.152	0.415	.714
rs12320939	T	G	0.484	Additive	−0.383	0.247	.122
				Dominant	−0.388	0.307	.206
				Recessive	−0.863	0.611	.159
rs6486795	T	C	0.378	Additive	0.225	0.204	.271
				Dominant	0.676	0.307	**.029**^∗^
				Recessive	−0.083	0.361	.818
rs7135617	G	T	0.414	Additive	−0.059	0.225	.792
				Dominant	−0.057	0.305	.852
				Recessive	−0.120	0.457	.794

### 3.3 Haplotype analysis

**Figure 2:**
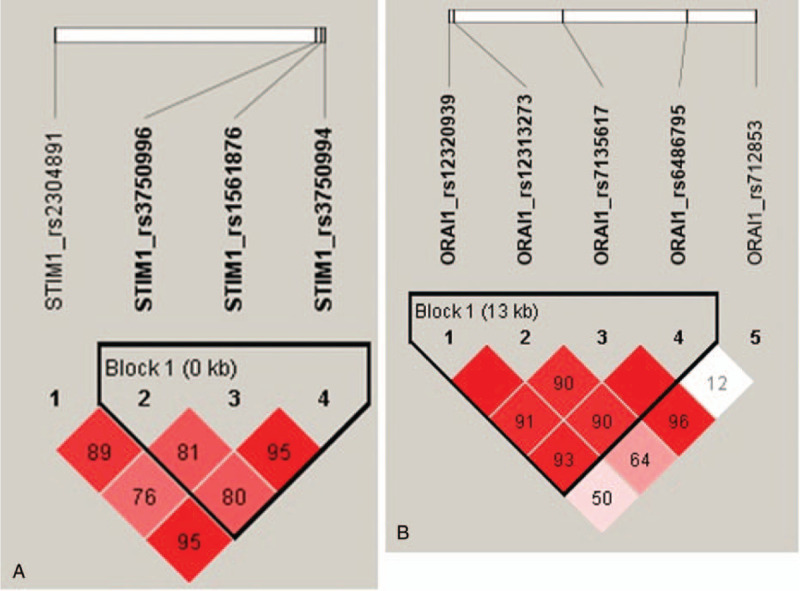
Linkage disequilibrium (LD) block of *STIM1* (A) and *ORAI1* (B) SNPs in Taiwanese ESRD patients based on D-prime (D’) value.

We further performed haplotype analysis to find more detailed information. The linkage disequilibrium blocks (pairwise D’ > 0.8) of *STIM1* and *ORAI1* are shown in Figure 2. Pairwise allele analysis revealed that *STIM1 *rs3750996/rs1561876/rs3750994 (A-A-T or G-A-T) showed a significant association with increased risk of EPO resistance (beta coefficients were 1.042 and 0.523), with a stepwise effect related to EPO resistance (Table 5). Meanwhile, *ORAI1 *rs12320939/rs12313273/rs7135617/rs6486795 with the allele combination of G-C-G-C had a significant association with EPO resistance (beta coefficient was 0.678), though it only presented a frequency of 0.010 and no consistent stepwise effect was found for other combinations (Table 5).

**Table 5:** Haplotype analysis of *STIM1* and *ORAI1* with the erythropoietin resistant index (ERI).

Haplotype	Freq^†^	Beta Coeff.^‡^	Std. Err.^§^	*P*-value^d^
*STIM1* (rs3750996/rs1561876/rs3750994)				
A/A/T	0.206	1.042	0.356	**.004**^∗∗^
G/A/T	0.496	0.523	0.262	**.046**^∗^
G/G/T	0.084	0.428	0.452	.344
G/G/G	0.195	Reference		
*ORAI1* (rs12320939/rs12313273/rs7135617/rs6486795)				
T/T/G/T	0.250	−0.517	0.300	.085
T/C/G/C	0.119	−0.067	0.292	.819
T/C/G/T	0.131	−0.386	0.363	.288
G/C/G/C	0.010	0.678	0.189	**.0003**^∗∗∗^
G/C/G/T	0.079	0.070	0.473	.883
G/C/T/T	0.386	Reference		

### 3.4 Functional annotation

We further assessed the functional characteristics and biological framework of the vatiants using bioinformatics analysis. As shown in the Supplemental Digital Content, Figure S1a and S1b, *STIM1* and *ORAI1* gene expression can be detected across different tissues. *STIM1* was abundantly expressed in skeletal muscle, and tibial artery, while *ORAI1* was abundant in skin, whole blood and spleen. Functional annotation showed that *STIM1* rs1561876 correlated with Ribonucleotide Reductase Catalytic Subunit M1 (*RRM1*) expression in skeletal muscle (*P* = 2.9 × 10^−9^, effect size = -0.21) (Table 6), with the AA genotype showing relatively low *RRM1* expression (Supplemental Digital Content, Figure S2a). Additionally, *ORAI1* rs6486795 was associated with *ORAI1* expression in whole blood (*P* = 1.3 × 10^−17^, effect size = 0.22), esophagus (*P* = 9.9 × 10^−16^, effect size = 0.29) and thyroid (*P* = 3.3 × 10^−13^, effect size = 0.23) (Table 6), with the CC genotype exhibiting higher *ORAI1* expression in whole blood (Supplemental Digital Content, Figure S2b). Using SNPNexus, we found that *STIM1* rs1561876 is a potential target for miRNA-939 (Table 7). Finally, from the Encyclopedia of DNA Elements (ENCODE) project, we found that rs1561876 is very likely to associate with the regulation of immune system via histone modifications.

**Table 6:** *cis*-eQTLs of ERI-associated SNPs in *STIM1* and *ORAI1* genes.

Gene	SNP	Genecode Id	Gene symbol	Tissue	*P*-value	Effect size
*STIM1*	rs1561876	ENSG00000167323.11	*STIM1*	Thyroid	1.30e-05	0.17
				Brain-Cerebellar Hemisphere	3.70e-05	0.39
		ENSG00000167325.14	*RRM1*	Muscle-Skeletal	2.90e-09	−0.21
				Artery-Tibial	1.10e-07	−0.21
				Artery-Aorta	3.50e-06	−0.26
				Adipose-Visceral (Omentum)	8.50e-06	−0.20
				Adipose-Subcutaneous	2.80e-05	−0.19
				Thyroid	3.40e-05	−0.15
				Esophagus-Muscularis	8.30e-05	−0.11
*ORAI1*	rs6486795	ENSG00000276045.2	*ORAI1*	Whole Blood	1.30e-17	0.22
				Esophagus-Mucosa	9.90e-16	0.29
				Thyroid	3.30e-13	0.23
				Stomach	7.60e-08	0.24
				Pancreas	4.50e-07	0.25
				Muscle-Skeletal	4.80e-07	0.11
				Colon-Transverse	1.50e-06	0.21
				Esophagus-Gastroesophageal Junction	2.90e-05	0.19
				Heart-Atrial Appendage	5.80e-05	0.17
				Esophagus-Muscularis	.00016	0.13

**Table 7:** Annotations of ERI-associated SNPs in *STIM1* and *ORAI1* genes.

Annotations^∗^	*STIM1* rs1561876	*ORAI1* rs6486795
Position	Ch11:4092165	Ch12:121638011
Location	3’-UTR	intronic
miRNA target sites	hsa-miR-939–5p (target site: 11:4092164-4092190)	N.A.^†^

ENCODE^‡^	Start	End	Class	Feature Type	Epigenome	Start	End	Class	Feature Type	Epigenome
	4080080	4092980	Histone	H3K36me3	Monocytes	121633920	121639380	Histone	H3K4me1	T cells
	4088120	4094680	Histone	H3K4me1	T cells	121634240	121642540	Histone	H3K4me1	NK cells
	4088220	4092800	Histone	H3K4me1	NK cells	121635580	121639040	Histone	H3K4me1	Monocytes
	4089180	4092680	Histone	H3K36me3	B cells	121635740	121639300	Histone	H3K4me1	B cells

## 4 Discussion

In this study, our results indicated that SOC-related genetic polymorphisms are significantly correlated with the risk of EPO resistance in dialysis patients. Two genetic polymorphisms rs1561876 and rs6486795 were identified here. Patients who carried the AA genotype of rs1561876 or CC/CT genotypes of rs6486795 have increased risk of EPO resistance. The results implied a need for higher doses of EPO in these patients. Functional annotation of eQTLs showed that the AA genotype of rs1561876 correlated with lower expression of *RRM1* in skeletal muscle. RRM1 is an essential enzymel for the conversion of ribonucleotides into deoxyribonucleotides.^[20]^ It is required for DNA replication during the S phase of the cell cycle, as well as DNA repair. The mechanism underlying the association of *RRM1* expression and EPO resistance remains unclear.

Identification for epigenomic parameters from database have become increasingly available in recent years, allowing researchers to understand the functions of non-coding polymorphisms at the epigenomic level.^[21,22]^ In this study, we found rs6486795 is located in an intron, however, this polymorphism may participate in the regulation of nearby genes by functioning as a transcriptional modulator. In addition, from the SNPnexus database, we demonstrated that rs6486795 is likely to involve in histone modifications which in turn affect gene expression in immune cells (T cells, B cells, natural killer cells). Thus, we presume rs6486795 is a regulator of inflammation, which is known to contribute to EPO resistance via increase of pro-inflammatory cytokines and suppression of erythroid progenitor proliferation.^[23]^

The downstream EPO signaling pathway has been studied regarding its pathogenic roles in EPO resistance. For example, a study from the U.K. Darlington research group found that EPO-activated JAK-STAT signaling pathway is blunted in patients with poor EPO response.^[24]^ Other downstream signaling pathways have been examined as well, including STAT5, PI3K, NF-κB, and Ras/MAPK.^[25–27]^ These studies indicated that the EPO signaling pathways may contribute to different EPO sensitivities. PLC-γ1 leads to PIP_2_ hydrolysis and IP_3_ generation, which activates the release of calcium from intracellular stores, and the intracellular calcium is sustained by extracellular calcium entry.^[28]^ EPO activation may contribute to the rise of intracellular calcium.^[28]^ Zhou XJ et al, showed that EPO reversed the attenuation of calcium surge in dysfunctional platelets of uremic rats, leading them to conclude that platelet dysfunction and defective calcium signaling may be improved by EPO therapy.^[29]^ Miller et al, reported that EPO induces a dose-dependent rises in intracellular calcium by a voltage-independent calcium channels.^[30]^ Based on these mechanistic studies and our findings, we presumed that SOCs involve in EPO sensitivity of patients. Indeed, recent work has shown that SOCs are associated with inflammation in several diseases. Wei et al reported that rs3750996/rs3750994 in *STIM1* are significantly correlated with higher inflammation in ankylosing spondylitis patients.^[31]^ Another strong piece of evidence comes from a demonstration that *STIM1* promotes colorectal cancer cell migration by increasing the expression of cyclooxygenase-2 and prostaglandin E2.^[32,33]^ In light of this mounting evidence, our functional annotation implied the possibility of SOC-mediated EPO resistance via inflammatory regulation.

While our study identified some important associations between genotypes and EPO resistance, there were several limitations in the study. For example, genotyping was conducted in a small number of patients. Additionally, although we made multivariable statistical adjustments, some unidentified clinical confounding factors may still influence EPO resistance, such as chronic inflammation, secondary hyperparathyroidism and pure red cell aplasia.^[34]^ Finally, large differences can exist in genotypic distribution between ethnicities, and our study was conducted in an ethnically homogeneous Taiwanese population. Based on these limitations, our findings should be confirmed with a larger multi-ethnic population and longitudinal study design.

## 5 Conclusions

Our study indicated novel pharmacogenomic associations of polymorphims in *STIM1* and *ORAI1* with the risk of EPO resistance. Functional annotations of the polymorphisms suggested that the underlying mechanism of calcium-dependent pathways, addressing further investigations into the roles of SOC polymorphisms in the regulation of inflammation and EPO signaling.

## Author contributions

CCK: study concept and design; analysis and interpretation of data; drafting of the manuscript; HSCW: analysis and interpretation of data; researched data; YJW: analysis and interpretation of data; WHC: analysis and interpretation of data; MSW: study concept and design; final approval of the version to be published; WCC: study concept and design; final approval of the version to be published.

**Conceptualization:** Chih-Chin Kao, Wei-Chiao Chang.

**Formal analysis:** Henry Sung-Ching Wong.

**Investigation:** Yu-Jia Wang, Wan-Hsuan Chou.

**Methodology:** Dyah Aryani Perwitasari.

**Project administration:** Dyah Aryani Perwitasari.

**Writing – original draft:** Chih-Chin Kao, Wei-Chiao Chang.

**Writing – review and editing:** Mai-Szu Wu, Wei-Chiao Chang.

## Supplementary Material

Supplemental Digital Content

## Supplementary Material

Supplemental Digital Content

## Supplementary Material

Supplemental Digital Content
