# Supplementary material for: The role of genetic polymorphisms in STIM1 and ORAI1 for erythropoietin resistance in patients with renal failure
Source: Medicine (Baltimore). 2021 Apr 30;100(17):e25243. doi: 10.1097/MD.0000000000025243 (PMC8083997; doi:10.1097/MD.0000000000025243)
Supplement: Supplemental Digital Content [file medi-100-e25243-s001.docx]

| **Supplementary table**  **Table S1.** Hardy-Weinberg equilibrium (HWE) test of selected tSNPs in *STIM1* and *ORAI1* | |
| --- | --- |
| SNP | *P*-value^a^ |
| *STIM1* |  |
| rs2304891 | 0.525 |
| rs1561876 | 0.463 |
| rs3750994 | 0.895 |
| rs3750996 | 0.204 |
| *ORAI1* |  |
| rs712853 | 0.087 |
| rs12313273 | 0.025 |
| rs12320939 | 0.054 |
| rs6486795 | 0.093 |
| rs7135617 | 0.065 |
| ^a^*P-*values were calculated by Chi-square test with continuity correction. | |
