# Supplementary material for: The role of genetic polymorphisms in STIM1 and ORAI1 for erythropoietin resistance in patients with renal failure
Source: Medicine (Baltimore). 2021 Apr 30;100(17):e25243. doi: 10.1097/MD.0000000000025243 (PMC8083997; doi:10.1097/MD.0000000000025243)

**Supplementary figure S1.** *STIM1* (a) and *ORAI1* (b) gene expression across different tissues. Vertical axis represents in transcripts per million (TPM). These data was obtained from the GTEx Portal (https://gtexportal.org/home/) on Mar 20, 2020.

(a)


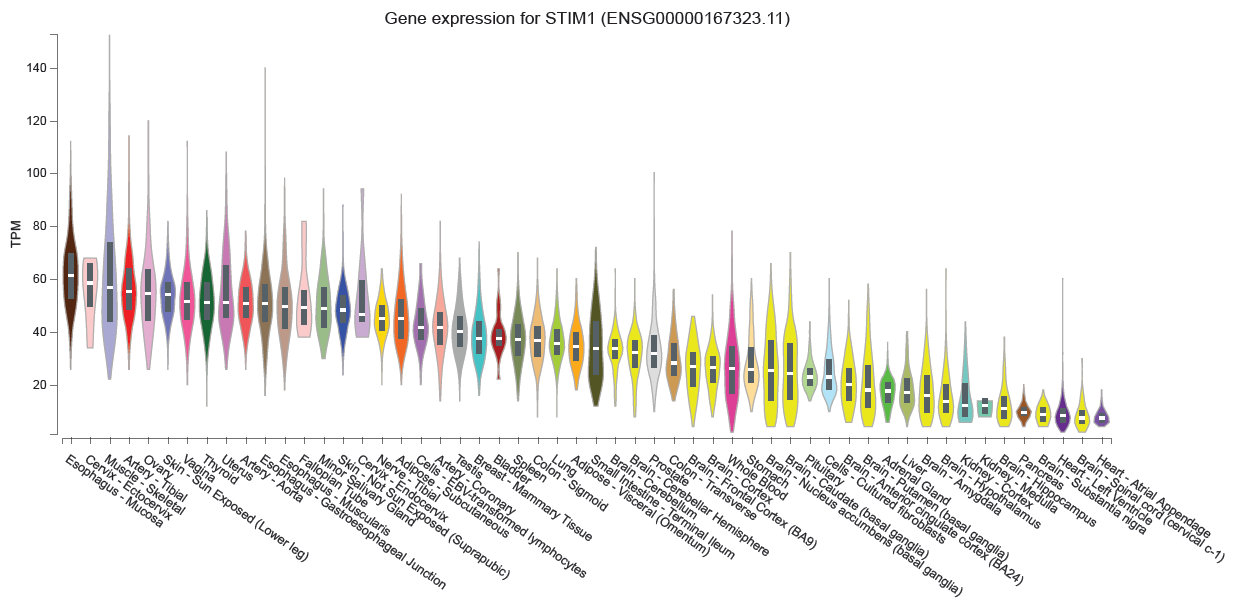


(b)


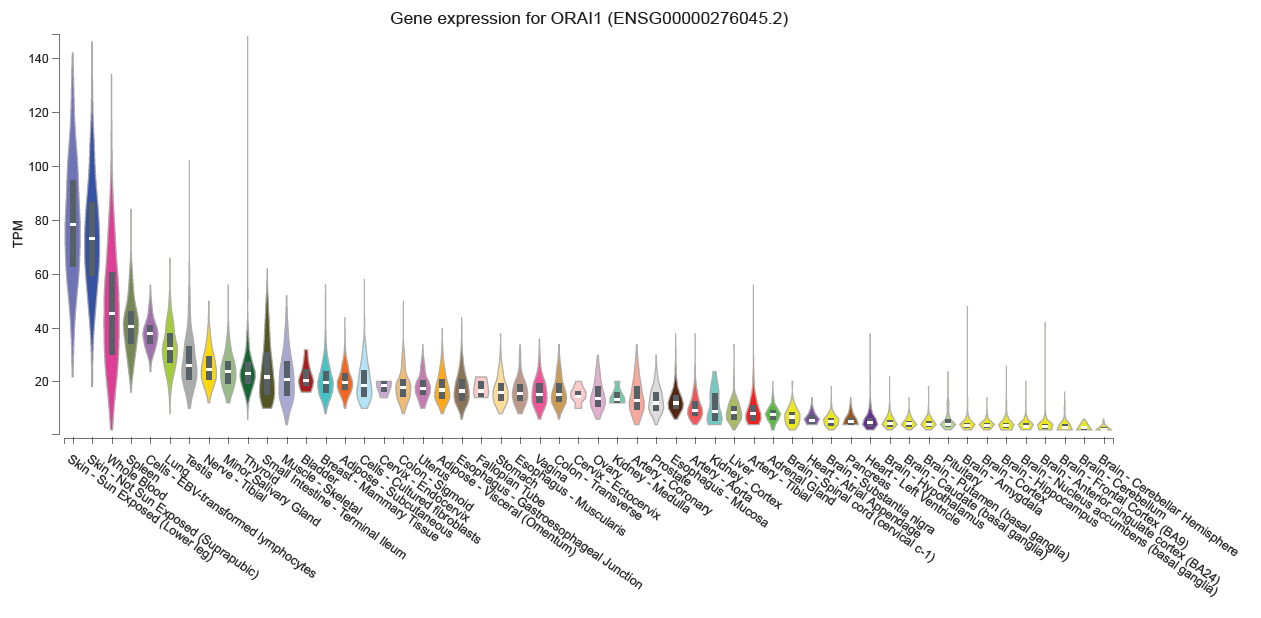

Supplement: Supplemental Digital Content [file medi-100-e25243-s002.docx]
