# Supplementary material for: The role of genetic polymorphisms in STIM1 and ORAI1 for erythropoietin resistance in patients with renal failure
Source: Medicine (Baltimore). 2021 Apr 30;100(17):e25243. doi: 10.1097/MD.0000000000025243 (PMC8083997; doi:10.1097/MD.0000000000025243)

**Supplementary figure S2.** eQTL box plot of rs1561876 in *STIM1* (a) and rs6486795 in *ORAI* (b) *1*. The AA genotype of rs1561876 had a relative lower expression of *RRM1* in muscle-skeletal tissue; the CC genotype of rs1561876 had a relative higher expression of *ORAI1* in whole blood

(a)


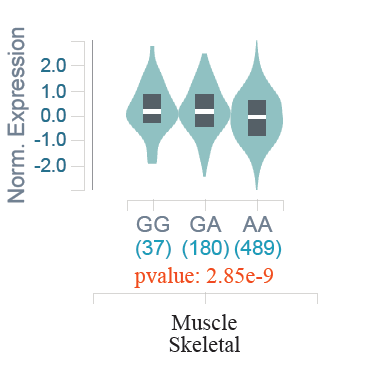


(b)


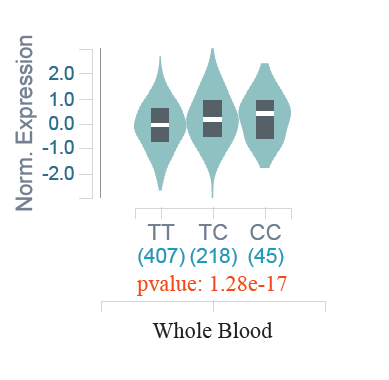

Supplement: Supplemental Digital Content [file medi-100-e25243-s003.docx]
